# Decolonizing Local Health: Advancing from Participation to Equitable Partnership along the Thailand–Myanmar Border

**DOI:** 10.5334/aogh.5418

**Published:** 2026-09-11

**Authors:** Myat Htoo Razak, Cynthia Maung

**Affiliations:** 1 School of Global Health, Faculty of Medicine, Chulalongkorn University, Bangkok, Thailand; 2 Mae Tao Clinic, Mae Sot, Thailand

**Keywords:** decolonizing local health, Thailand–Myanmar border, ethnic health organizations

## Abstract

*Background:* The movement to decolonize global health has increasingly challenged inequities in funding, leadership, knowledge production, authorship, and representation between global and local actors. However, less attention has been given to power dynamics within local and national health systems and partnerships. We propose extending the decolonization lens to **local health:** examining who leads, decides, speaks, controls resources, produces knowledge, and benefits within health systems themselves.

*Perspective:* Drawing on decades of experience along the Thailand–Myanmar border, we reflect on the evolution of ethnic health organizations (EHOs), community-based health systems, and their partnerships with donors, governments, international organizations, and intermediary organizations. The border provides a distinctive setting for this discussion: over several decades of conflict, displacement, migration, and political instability, locally developed and sustained health systems have delivered essential services to underserved populations and increasingly assumed roles in health workforce development, health information, disease control, and emergency response. These experiences demonstrate substantial progress toward locally led health systems, but also persistent asymmetries in funding, agenda setting, knowledge production, representation, and risk sharing. In particular, localization of implementation may advance without equivalent transfer of decision-making power.

*Implications:* Decolonizing local health requires moving beyond participation toward equitable partnership. Funding mechanisms should enable greater direct and flexible support to local organizations; partnerships should share governance and decision-making; local actors should receive equitable opportunities for authorship and representation; and accountability and risk should be shared more fairly. The experience of the Thailand–Myanmar border suggests that decolonization is not only about shifting power between global and local actors, but also about redistributing power among actors within local health systems. Ultimately, decolonization should be judged not by whether local actors are invited to participate, but whether they are trusted and empowered to lead.

The call to decolonize global health has gained increasing attention in recent years [1–3]. Much of the discussion has focused on shifting power from institutions in high-income countries (HICs) to actors in low- and middle-income countries (LMICs), raising important questions about funding, leadership, authorship, and representation [4–6]. Yet decolonization is often more complex than a transfer of power from the Global North to the Global South.

Our experience working along the Thailand–Myanmar border for decades has led us to a related question: **What does it mean to decolonize local health?**

The border region has long been shaped by wars, conflicts, displacement, diverse ethnic groups, migration, and limited access to formal health services. The military coup in Myanmar in 2021 has triggered large-scale new displacement, increased attacks on health facilities, and further deterioration of health services. In response, ethnic health organizations (EHOs), community-based organizations (CBOs), faith-based groups, local civil society organizations (CSOs), and international partners with support from Thailand have built a diverse health ecosystem serving hundreds of thousands of people annually [7, 8].

This experience reveals both the possibilities and limitations of decolonization. Progress has been made in recognizing local expertise and expanding participation. Yet challenges remain regarding who controls resources, shapes agendas, owns knowledge, and represents communities in decision-making processes. The central lesson from the border is that decolonization is fundamentally about who leads, who decides, who speaks, and who benefits from available resources.

## Decolonizing Local Health

The dominant discourse on decolonizing global health often focuses on relationships between actors from HICs and LMICs. While important, this perspective can overlook power dynamics within countries and affected areas.

Decolonizing global health asks how power is redistributed between global and local actors; decolonizing local health asks how power is distributed among the actors within local health systems themselves.

The Thailand–Myanmar border is distinctive not merely because it is conflict-affected, but because decades of conflict and displacement have produced a locally developed and sustained cross-border health ecosystem in which EHOs and community actors have built and sustained parallel health-system functions while negotiating continuously with governments, donors, international organizations, different ethnic leaderships, and intermediary partners [9, 10].

Power does not flow along a single axis in the border. In some cases, authority shifts from international actors to intermediary organizations without involving communities themselves. In others, local organizations play major implementation roles while remaining excluded from funding decisions and strategic leadership.

Decolonizing local health therefore requires examining whether communities and organizations closest to health challenges have meaningful influence over decisions affecting their lives.

## Progress and Persistent Challenges

The border experience offers reasons for optimism. Over the past two decades, EHOs have evolved from being viewed primarily as humanitarian actors into recognized health system partners [11–13]. Their contributions to service delivery, workforce development, health information systems, disease control, and emergency response are increasingly acknowledged by researchers, donors, and policymakers [14–17].

Community health workers, local clinics, and ethnic health systems have demonstrated remarkable resilience through military coups, conflicts, displacement, public health emergencies, and political instability. Their success has challenged assumptions that expertise and leadership from external institutions are essential.

Partnerships have also improved. Compared with earlier humanitarian models, many organizations now emphasize consultation, risk sharing [18], local participation, capacity strengthening, and shared planning. Increasing numbers of local health leaders contribute to technical working groups, policy discussions, and regional forums.

However, important challenges remain. One recurring concern is that localization of implementation has progressed faster than localization of power. Local organizations may deliver services, collect data, and maintain community relationships, while decisions about funding, priorities, knowledge production, and public representation remain concentrated elsewhere. Although community organizations generate valuable evidence and operational knowledge, they do not always receive equitable recognition, authorship, or representation in conferences and policy discussions. Consequently, the voices of those closest to health challenges may remain underrepresented in spaces where decisions are made.

These challenges are reinforced by institutional incentives and partnership structures [19]. Organizations and individuals are often rewarded for securing grants, producing publications, and increasing institutional visibility. Local health organizations are frequently expected to align proposals and programs with donor and intermediary priorities, sometimes at the expense of responding to rapidly changing local realities and community-defined needs.

Local partners often bear a disproportionate share of operational, security, compliance, and reputational risks through extensive reporting requirements and liability arrangements. Together, these dynamics can constrain local ownership and leadership despite widespread commitments to localization and equitable partnership. The question is not whether organizations benefit from partnerships, but whether communities benefit proportionately from the resources, recognition, and opportunities generated in their names.

**An intermediary paradox.** In some border partnerships, organizations positioned between donors and EHOs have played an important role in facilitating funding, technical support, and access to international networks. These arrangements can be valuable, particularly when local organizations face legal, financial, or administrative barriers to receiving international funds directly. Yet they can also create new forms of dependency.

Staff of intermediary organizations who possess the legal status, documentation, language skills, and mobility required to work and travel internationally may receive substantially greater access to institutional resources and professional opportunities than frontline health workers whose services and data sustain the partnership. Local organizations may also generate much of the operational knowledge and data subsequently used in reports, presentations, publications, or academic work led by intermediary staff, while receiving limited authorship, recognition, or professional benefit. Because continued access to funding may depend on the intermediary relationship, local partners may have limited ability to challenge these arrangements openly. Thus, an arrangement intended to facilitate localization can inadvertently reproduce the very power asymmetries it seeks to overcome.

This illustrates why decolonization cannot be assessed simply by asking whether an organization is locally registered or whether local people are involved in implementation. The more important questions are: Who controls the resources? Who benefits from the knowledge generated? Who receives recognition? Who has the freedom to speak? And who can challenge the partnership without jeopardizing access to essential funding?

Localization of implementation is not the same as localization of power. An organization can be local in nationality, but not local in power.

## From Participation to Equitable Partnership

The next phase of decolonization should focus on moving from participation to equitable partnership. Participation is important, but communities can participate extensively without exercising meaningful influence. Equitable partnership means that local actors help set priorities, control resources, generate and own knowledge, represent themselves, and hold partners accountable.

Equitable partnership does not require the absence of international actors. Donors, multilateral agencies, universities, and NGOs continue to play important roles. Their success, however, should increasingly be measured by the extent to which they strengthen local leadership rather than maintain dependence on external support.

Five actions can help advance this transition:

1. **Reform funding mechanisms** to increase direct support for EHOs and CSOs by simplifying grant eligibility and channel direct funding to EHOs and CSOs through reducing unnecessary reliance on external fiscal sponsors.
2. **Co-design programs and governance structures** that share decision-making authority, responsibility, and accountability, while strengthening trusts among EHOs, CSOs, and intermediary organizations.
3. **Promote meaningful representation** by supporting local leaders to participate directly in decision-making process and national/global forums, including allocating funded seats and speaking roles for local leaders with support for travel, visa facilitation, and fair remuneration.
4. **Ensure equitable authorship and knowledge ownership**, enabling local practitioners to lead publications and presentations by establishing collaborative authorship agreements from the beginning of the projects/activities.
5. **Strengthen accountability to communities** through transparent decision-making, community feedback mechanisms, and accessible communication in local languages on performance metrics and public reporting on financial and strategic decisions.

Investing in leadership development, mentorship, and communication skills for emerging local leaders is equally important, in addition to addressing legal, language, and travel barriers. Those closest to health challenges should be able to share their experiences and insights directly and confidently with policymakers, researchers, and donors.

## Conclusion

The experience of the Thailand–Myanmar border suggests that decolonization is an ongoing process. Over the past decades, EHOs, community-based health systems, and their partners have demonstrated that locally led approaches can deliver essential services, build resilience, and respond effectively to conflict, displacement, and public health emergencies. Their role has become even more critical amid the worsening crisis since the 2021 coup.

The next phase of decolonization must move beyond participation toward equitable partnership. Progress should be measured by whether communities and organizations closest to health challenges have the authority, resources, recognition, and opportunities to shape their own futures. Ultimately, the test of decolonization is not whether local actors are invited to participate, but whether they are trusted and empowered to lead.

*Myat Htoo Razak and Cynthia Maung*

## Data Availability

Both authors had access to the data and a role in writing this manuscript.
